# New understanding of primary health care nurse practitioner role optimisation: the dynamic relationship between the context and work meaning

**DOI:** 10.1186/s12913-019-4731-8

**Published:** 2019-11-21

**Authors:** Nancy Côté, Andrew Freeman, Emmanuelle Jean, Jean-Louis Denis

**Affiliations:** 1 Faculty of Social Sciences, Université Laval, Quebec City, Canada; 2 Centre de recherche sur les soins et les services de première ligne de l’Université Laval (CERSSPL-UL), Quebec City, Canada; 3 Department of Rehabilitation, Faculty of Medicine, Université Laval, Quebec City, Canada; 4 Department of Nursing Sciences, Université du Québec à Rimouski, Rimouski, Canada; 5 Département de gestion, d’évaluation et de politique de santé, École de santé publique, Université de Montréal, Montréal, Canada; 6 Chaire de recherche du Canada sur la transformation, le design et l’amélioration des systèmes de santé, Montréal, Canada

**Keywords:** Nurse practitioners, Primary health care nurse practitioners, Delivery of health care, Optimisation of health professional role, Work meaning

## Abstract

**Background:**

Optimising health professionals’ contribution is an essential step in effective and efficient health human resources utilisation. However, despite the considerable efforts made to implement advanced practice nursing roles, including those in primary care settings (PHCNP), the optimisation of these roles remains variable. In this investigation, we report on the subjective work experience of a group of PHCNPs in the province of Quebec (Canada).

**Methods:**

We used Giddens’ structuration theory to guide our study given its’ facilitation of the understanding of the dynamic between structural constraints and actors’ actions. Using a qualitative descriptive study design, and specifically both individual and focus group interviews, we conducted our investigation within three health care regions in Quebec during 2016–2017.

**Results:**

Forty-one PHCNPs participated. Their descriptions of their experience fell into two general categories. The first of these, their perception of others’ inadequate understanding and valuing of their role, included the influence of certain work conditions, perceived restrictions on professional autonomy and the feeling of being caught between two professional paradigms. The second category, the PHCNPs’ sense of engagement in their work, included perspectives associated with the specific conditions in which their work is situated, for example, the fragility of the role depending on the particular clinic/s in which they work or on the individuals with whom they work. This fragility was also linked with certain health care reforms that had been implemented in Quebec (e.g., legislation requiring greater physician productivity).

**Conclusion:**

Several new insights emerged, for example, the sense of role fragility being experienced by PHCNPs. The findings suggest an overarching link between the work context, the meaning attributed by PHCNPs to their work and their engagement. The optimisation of their role at the patient care level appears to be influenced by elements at the organisational and health system context levels. It appears that role optimisation must include the establishment of work environments and congruent health context structures that favour the implementation and deployment of new professional roles, work engagement, effective collaboration in interprofessional teams, and opportunities to exercise agency. Further research is necessary to evaluate initiatives that endeavour to achieve these objectives.

## Background

The worldwide need for highly skilled healthcare workers is increasing [1] and the underutilisation of health human resources is a significant social problem [2]. Strategies aimed only at increasing the numbers of healthcare professionals may not address all deficiencies of healthcare services [3]. Integrating and optimising the contribution of healthcare professionals, including ensuring that they are able to work to their full scope of practice, is perhaps one of the most critical actions that healthcare services are undertaking internationally [4, 5].

In one of the responses to these challenges, advanced practice nursing (APN) roles continue to be developed and used internationally [6]. Although some variability exists in how this role is defined [7], the International Council of Nurses [8] defines a Nurse Practitioner/Advanced Practice Nurse as “a registered nurse who has acquired the expert knowledge base, complex decision-making skills and clinical competencies for expanded practice, the characteristics of which are shaped by the context and/or country in which s/he is credentialed to practice”.

In Canada, two types of APN roles are recognized: Nurse practitioners (NP) and Clinical Nurse Specialists. Only NP titles are protected. All provinces and territories in Canada have legislation and regulations for NPs [9]. From 2006 to 2019, NPs in the province of Quebec were the only ones in Canada not permitted to diagnose, communicate a diagnosis, or initiate treatment for a chronic disease. Paradoxically, Quebec NPs receive the most hours of theoretical and clinical (internship) training. Work is currently underway to harmonize pan-Canadian regulatory frameworks. In Quebec, long-term collaboration between the *Ordre des Infirmières et Infirmiers du Québec*, the *Ministry of Health and Social Services* and the *College des Médecins du Québec* led to the tabling in 2019 of Bill 43 (*Loi modifiant la Loi sur les infirmières et les infirmiers et d’autres dispositions afin de favoriser l’accès aux services de santé*). If passed, this law will authorise Quebec NPs, according to their specialty class and area of care, to diagnose certain diseases.

In Quebec, NPs currently work in five specialties: adult care, paediatric care, mental health, neonatology, and primary health care. Our focus in this article concerns this latter group, the Primary Health Care Nurse Practitioners (PHCNP). In Quebec, the PHCNP role was introduced in 2007 with the intention of improving the accessibility of care and front-line services [10]. The creation of 500 PHCNP positions was announced in 2010 to favour its’ implementation [11]. Subsequently, in 2014, the Quebec government announced that the health network could count on the availability of 2000 extra APNs by 2025 for primary care and other practice sectors [12]. In light of this significant commitment, it is essential to understand the conditions necessary for the optimal use of this role.

Several studies have documented barriers and facilitators that influence the implementation of PHCNPs; it can be difficult to distinguish these given the possibility that barriers can be facilitators in some contexts and vice versa. Some of the factors that have been identified include: reimbursement policies [7, 13–15]; scope of practice clarity [7, 14, 16]; role definition, comprehension and recognition [7, 13, 14, 17–20]; integration process into primary care settings [21, 22]; organisational/administrative support [7, 13, 14, 18, 20, 23, 24]; work climate [25]; team functioning in general [26] and collaboration between APNs and physicians [13, 17, 19, 27–29] and with registered nurses and other professionals [16, 17, 19]. Studies have demonstrated the positive impact associated with adopting an systematic process to implementing the PHCNP role [19, 21]. In a related vein, others have noted the importance of considering multiple levels (e.g., organisational, health care system) in better understanding the elements needed for an optimal implementation [3, 16, 30, 31].

Various theoretical approaches have been used in efforts to understand how to optimise the use of PHCNPs, These approaches have included, for example: contingency theory [26], organisational levels of support practices [31], organisational change management framework [32], comprehensive conceptual model [3], a combined logic and implementation analysis [21], and deliberative dialogue [33]. A *boundary work* framework also was proposed, albeit within an acute care setting [34]. However, despite the useful insights gained to date regarding the integration and deployment of PHCNP roles, including the positive links found with patient health results, satisfaction with care, health care costs and even system improvements [35–38], the PHCNP foothold in general practice remains variable [39]. This inconsistent success suggests that further understandings are required.

Although some attention has been paid to identifying and describing multiple factors (e.g., work organisation dimensions; physician remuneration), limited attention has been given to the dynamic interaction between the individual, the organisation and the broader health system context [16, 19, 30, 31]. The interdependence between these different levels can shape a particular context that evolves over time and is a source of both constraints and possibilities.

In this light, we have used Giddens’ [40] structuration theory to guide our investigation because it facilitates understanding of the dynamic between structural constraints and actors’ actions. This theory takes into account four dimensions: (1) The structural, that is, the rules and resources that define organisations or social systems (e.g., Quebec health system); (2) the actions taken by the actors within these organisations; (3) the intentionality of these same actors, either the rationale or the motivation at the source of their actions; and (4) the spatio-temporal context. According to this theory, the *structural* presents a duality: it can be both constraining (setting limits to the actions of actors) and enabling (gives actors the *skills* to take action). In the context of our study, using this theoretical framework means taking into consideration how structures, embodied in rules and resources within a given health system, might constrain the ability of PHCNPs to exercise their role in an optimal way. It also leads us to pay attention to the active role that the PHCNPs might play in appropriating these rules and resources in order to act on the constraints of their practice. As well, this framework leads us to analyze different dimensions of the organisation of work of the PHCNPs at the individual, the organisation and the broader health system context levels. Ultimately, this theoretical approach would provide a basis for better understanding the variability in the optimisation of the PHCNP role within health systems and organisations.

In this article, we report on the subjective work experiences of a group of PHCNPs in the province of Quebec (Canada). By subjective work experience, we are referring to how these professionals experience and enact their role relative to the contexts within which their practice is situated. This understanding is necessary to create work environments and other contexts (e.g., Ministry of Health) that promote the optimal use of the role. The findings that we report in this article are one component of our global investigation, the objective of which was to better understand the factors that impede or facilitate local, regional and provincial stakeholders in achieving an optimal use of the PHCNP role.

## Methods

### Study design

We used a qualitative descriptive study design [41], guided by Giddens’ theory, to empirically probe the dynamics between the factors that influence PHCNP role optimisation..

### Study sample and participants

We conducted our investigation during 2016–2017 within three health care regions in Quebec due to their distinguishing characteristics (population, geographic location, services, administrative structures) and diverse conditions in which the PHCNP role was being implemented. There were 20, 6 and 25 PHCNPs respectively working in each of these three regions. This variability is consistent with the respective populations of people in these regions.

We conducted our investigation across a range of demographic (e.g., urban, semi-urban, rural) and administrative [community health clinics; family medicine practices of various configurations (e.g., university affiliation, drop-in clinics)] structures. To participate in the study, the PHCNPs were required to have worked in this role for a minimum of 6 months. We used a maximum variation sampling approach [42] to recruit these professionals in order to capture the diverse clinical settings in which they practise. In the three regions in which our investigation was conducted, we informed the PHCNPs about our study via their provincial professional association (*Association des infirmières praticiennes spécialisées du Québec*), and specifically via the regional representatives, who invited those individuals who were potentially interested to contact us for more information.

We created two advisory committees to support the rigour of our study by means of their expertise about the PHCNP practice context and current issues being faced by different stakeholders in the implementation of the PHCNP role. These committees provided advice regarding recruitment and data analysis. See Table 1 for the composition of these committees.

**Table 1:** Composition of advisory committees

Type of committee	Members
*Strategic committee*	1 representative of the Quebec physicians’ professional licensing board
	1 representative of the Quebec nurses’ professional licensing board
	2 representatives from the Quebec Ministry of Health and Social Services
	The Director of Nursing Services (DNS) from the *Centre intégré universitaire de santé et de services sociaux de la Capitale-Nationale* (CIUSSS-CN)
	The Director of Professional Services (DPS) from the CIUSSS *de la Capitale-Nationale*
	The president of the Quebec association of PHCNPs
*Work committee*	1 family physician with experience working with PHCNPs
	1 PHCNP from each of the three regions
	2 regional directors of general medicine
	1 regional DNS and 1 assistant DNS
	1 regional DPS
	1 patient partner recruited by means of the *Strategy for Patient-Oriented Research*

### Data collection

We used both in-depth semi-structured individual interviews and focus group interviews. The respective strengths of these two approaches [42] permitted the emergence of a richer and more trustworthy understanding of the PHCNPs’ subjective work experience. Given the potentially sensitive nature of the questions, the individual interviews were appropriate for understanding the subjective work experience of the PHCNPs, their perceptions of their role and how it was being used within multidisciplinary teams. In turn, the focus group interviews, because of the exchanges among participants, were appropriate for deepening understanding and exploring potential avenues for optimising the PHCNP role.

The individual interview guide reflected Gidden’s theoretical approach. For example, we sought to understand how the PHCNPs’ work was being experienced (motivations and actions) as well as the resources (what was needed, available or lacking). However, the open-ended questions also permitted the participants to discuss other elements that they considered relevant. Specifically, the individual interview guide included the four following principal dimensions: (1) the reasons for choosing to become a PHCNP; (2) the nature of their experience as a PHCNP, including their integration into the team and the evolution of their experience; (3) the use of their role in teams and any actions taken to increase their ability to exercise their role to its fullest extent; and (4) the resources and support necessary for the exercise of an optimal practice. The individual interviews (average 1.5 h duration) were conducted either face-to-face or by Skype, as requested by the participants.

In turn, the focus group interviews (average 2.25 h duration) were conducted following the initial analysis of the individual interview data. In these interviews, we further explored the impact of the conditions in which the PHCNPs exercised their role on how they viewed the meaning of their work. Specifically, we focused upon three themes that emerged from the individual interviews and that appeared to be significant for PHCNPs’ subjective experience: (1) the integration and deployment of their role, (2) the optimization of teamwork, and (3) work meaning. We also pursued questions about the resources that they believed they needed to carry out their work, and potential solutions that would help them to optimise their role.

Participants provided written consent to participate in our investigation. As well, with the participants’ consent, both the individual and focus group interviews were audio-taped.

### Data analysis

The audio-taped individual and focus group interviews were transcribed and anonymised. We used the data analysis process outlined by Miles and Huberman [43]. Although the data analysis was deductive in the sense that its’ starting point was the interview structures that were guided by Gidden’s theoretical approach, the subsequent analysis was inductive. For both the individual and focus group interviews, a comprehensive summary of each interview was prepared; these summaries were structured according to the interview guide elements and the themes that emerged. This coding was carried out by the first author and two research professionals, using the NVivo software, to permit greater interrater reliability. Subsequently, a matrix was constructed to organise the themes as they emerged; this information constituted the first level of analysis. Over the course of the investigation, the analysis of the individual and focus group interview data was regularly discussed with the other researchers. As well, the emerging findings were presented to the members of both advisory committees. These members’ questions and reflections were used to clarify the analysis of the data.

Consistent with the inductive and iterative data analysis process that we used, the data collection and analysis steps occurred simultaneously. This approach was also consistent with our intention to achieve data saturation. That is, as our understanding of the findings gradually emerged, we used subsequent interviews to pursue these impressions in endeavouring to arrive at a strong understanding of the phenomenon.

In summary, consistent with qualitative inquiry, we adhered to several criteria to create authenticity in our investigation [44], including: inductive data analysis, analysis records (e.g., decision trail, decision rules), audio taping/verbatim transcription for content, data saturation, accuracy, peer audit to confirm coherence (using the range of disciplines of the research team: sociology, nursing, rehabilitation, policy analysis), ongoing discussion with the members of the *Strategic committee* and the *Work committee*, and participants’ actual quotations to provide *thick* description of their experiences.

## Results

### Participants

A total of 27 PHCNPs participated in the study. Sixteen PHCNPs participated in individual interviews and 25 participated in one of three focus group interviews; 14 PHCNPs participated in both individual and focus group interviews. See Table 2 for the participant details by region.

**Table 2:** Participants

Region	Total number of PHCNPs in region	Practice experience	Individual interviews	Focus group interviews
1	20	Between 9 months and 7 years	8	8
2	6	Between 13 months and 6 years	3	6
3	25	Between 15 months and 2 years	5	11
Total			16	25

Data analysis revealed that the subjective work experience of PHCNPs interviewed in this study fell into two general categories: (1) their perceptions regarding the understanding and valuing of their role and (2) their sense of engagement in their work. The themes that emerged from the individual interviews and focus group interviews are essentially the same. However, work meaning, work engagement and the feeling of fragility depending on the environment in which working are themes that were mainly discussed during the focus groups.

### Understanding and valuing of the PHCNP role

The specific perspectives that are linked to this category include the influence of certain work conditions, PHCNPs’ perceived restrictions on their professional autonomy and the feeling of being caught between two professional paradigms.

### Work conditions

The PHCNPS reported two elements associated with the conditions in which they practised that they perceived as indicative of an inadequate understanding and valuing of their role. First, the majority of participants reported that their large workload frequently obliges them to work overtime hours, either by prolonging their work day or by taking work home to be completed in the evenings or on the weekends. They are generally accepting of doing these overtime hours, believing them to be unavoidable and inherent within their role. However, they feel dissatisfied that these extra hours are not remunerated and that the measures in place for them to compensate for this time are not adapted to the reality of their role. The refusal to pay them for the overtime hours, in combination with a salary that many PHCNPs believe to be insufficient, makes them feel that their role is under-recognised and undervalued. As noted by one participant (#01):

“I made a big discovery this year. I’m paid 35 hours per week; I estimate that my workload is around 500 patients for which I’m solely responsible, plus 300 shared patients, who are not simple cases. Last year, I worked around 60 hours a week … there are lab results that arrive, there are ultrasound results that arrive, I have to talk with my physician partners, there are emergencies that arrive. You have no life!”

The second element concerns many participants’ perception that their inflexible work schedule is not appropriate for their role. This schedule is not congruent with their responsibilities, which are actually more similar to those of physicians than those of registered nurses, with respect to the completion of patients’ records and requests for specialist tests or lab results, among other examples. As noted by a participant (#02), “We’re stuck in the straitjacket of our collective agreement; seven hours a day, no overtime, and so on.”

Both of these elements are associated to some degree with the collective agreement within which the PHCNPs’ work conditions are situated. As one participant (#05) noted, “For sure we have a … unionised role; however, nothing is adapted for us in the collective agreement”.

### Professional autonomy

The participants identified two main sources of frustration regarding restrictions on the professional autonomy within their scope of practice. In the first instance, the dissatisfaction for some PHCNPs is associated with the limits experienced in the context of their collaboration with their physician partners. Specifically, the PHCNPs felt that some physicians were preventing them from being able to practise to their full scope; that is, their role was being restricted to primarily meeting physicians’ requirements rather than fully exercising their role in a way that was helping meet the goal of improving patients’ access to quality primary care services. As noted by one participant (#03), “So where is the added value [of my role]? I feel like I’m in a survival mode, of protection, to try and protect what I’ve built.”

In the second instance, the dissatisfaction experienced by some PHCNPs is associated with their perception that their scope of practice is unnecessarily restrictive, specifically with respect to being permitted to diagnose and to initiate treatments for chronic diseases. The PHCNPs believed these two elements to be key components within their professional identity within the context of the evolution of their scope of practice that had taken place in Quebec. Within this context, these professionals also wondered about the specificity of their role relative to that of their registered nurse colleagues given these latter professionals’ recently acquired right to prescribe. “It’s like we’re caught. We’re stuck in a straitjacket that has been defined more for roles with less accountability, less unpredictability, less responsibility.” (#03).

These sources of dissatisfaction seemed to become more clear following the accumulation of a degree of experience in this role that occurs during the initial, approximately 18-month, integration phase. This phase is a period of adaptation for these professionals and includes elements such as appropriating their role and becoming familiar with their new clinical setting in general, with the procedures in place, with the services offered, and with their colleagues.

### Being caught between two professional paradigms

The third perspective identified by the PHCNPs with respect to the understanding and valuing of their role is that of their perception of feeling pulled between two professional paradigms, that is, the *caring* paradigm underlying nursing versus the biomedical approach underlying medicine. On the one hand, as nurses, they privilege a global health approach that takes into account physical, psychological and social dimensions. This role is synonymous with a prevention and health promotion approach within which a proactive educational role is intrinsic. On the other hand, some PHCNPs feel perceived by family physicians to be the equivalent of medical residents (*mini-doctors*). Among the consequences of this latter perspective is their experience of significant pressure regarding the number of patients that they should see. This pressure can translate into more of a caseload-centred (number of patients to be seen) versus patient-centered (customising the care to each patient’s needs) approach. This increased pressure was seen to be associated to some degree with the enactment in the province of Quebec in 2015 of Law 20 (*Loi favorisant l’accès aux services de médecine de famille et de médecine spécialisée*), which includes strong productivity expectations on physician practice. Although the PHCNPs recognise that a certain juggling of nursing and biomedical paradigms is an innate part of their professional role, they experience significant tensions in their practice when the nursing orientation is minimally or not acknowledged. As explained by one participant (#01):

We’re compared both by physicians and by registered nurses; neither group understands [what we do]. [I’m asked] « Why haven’t you gone to lunch yet? » Well, I haven’t got time. « What do you think I should do? I have to finish what I’m doing. If I don’t do it now, I’ll have to do it later. So I might as well get it done now. » I can’t put it off until tomorrow, because tomorrow, there will be another pile. The [registered] nurses, they don’t get it either. How many times have they said to me « Come on, what are you doing? ». But they don’t have my obligations … nobody seems to understand.

In this first section, the findings have revealed that the limits experienced by the PHCNPs in their daily practice appear to be associated with factors at the organisational and health care context levels. As will be seen in the next section, the rules and resources in place at these two levels shape the actions taken by the PHCNPs (actors) with respect to their sense of engagement in their work.

### PHCNPs’ sense of engagement in their work

The participants described several elements that influence their sense of engagement in their work. Some of these perspectives are associated with specific conditions in which these professionals’ work is situated. For example, several PHCNPS commented upon the impact of the fragility of their role that they experience depending on the particular clinic/s in which they work (milieu-dependent) or on the individuals with whom they work (individual-dependent). PHCNPs’ experiences vary widely, contributing to difficulties for them to fully engage in their work. It is as if they can never be quite sure to what extent they will be able to fully exercise their role and that the situation can change quickly. As one participant (#04) noted:

[It’s] the disappointment and the risk of what we are going to do with the profession, as a result of a change. It’s not a question of anxiety and then of fear of the unknown, it is the difficulty of finding a team that wants you to be there for the right reasons. And we don’t find these teams everywhere. Thus, from the time that our team falls apart, well, we know very well that the likelihood of finding another team like this is pretty slim, which makes us not feel very hopeful.

On a similar note, a number of PHCNPS commented upon the impact of the fragility of their role associated with health care reforms. As summed up by one participant (#05), “We are in the whirlwind of change, and we have no control over what is happening.” Among other elements, the participants are referring here to the increased productivity pressures on family physicians ensuing from the enactment in Quebec of *Loi 20*, which led to a restructuring of how some clinics functioned. As noted by a participant (#03):

The physician is at the center, he wants to see as many patients as possible, and he wants to surround himself with all the people who will help him achieve his goal … [This change has had] a negative impact on my partnerships, in the sense that there is a lot of energy being put into reorganise things for which my opinion is not sought. It is as if, in order to achieve the objectives, the new model is a physician with an auxiliary who sees all the patients before, then sees many, many, many [patients], then who does all that the physician asks him at the technical level, in order to go faster.

Subsequent to these and other similar types of situations, several PHCNPs spoke about their questioning of their future in the profession. As one participant (#01) specified:

It’s not enjoyable; there isn’t the same atmosphere. I’ve been here for 5 years but this is the first year that I’m not hopeful that things will improve. I’m asking myself “What am I doing?” I see other positions advertised but I ask myself if it’s even worth it to change. Is the grass greener on the other side? What should I do? I’m not going to put up with 20 years like this.

A number of participants spoke about being in a survival mode, for example: “Actually, I survive by taking it day by day. That’s how I see it. Each day, I’ll go to work.” (#01). As noted by another participant (#03), “I find myself in a survival mode, of protecting myself, in order to protect what I have built … I need to tell myself that’s it’s temporary and that things will improve. If they don’t, I won’t be able to hang on.”

Similarly, several PHCNPs indicated that they were finding it difficult to see a future in this profession. As noted by a participant (#06), “What I find frustrating is that at the start, I really had the fire; I still have it but sometimes, it’s true that after these three years, I envisage other perspectives. I’m willing to fight in order to eventually have better conditions, but not to the detriment of my health.”

Maternity leave was identified by some PHCNPs as an opportunity (*loophole*) to take time out from the difficult conditions to reflect upon their professional future. As noted by a participant (#07): “I’m on maternity leave but I doubt that I’ll return to the same job.”

## Discussion

Some aspects of the subjective experience of work described by PHCNPs in our study regarding barriers to the optimisation of their role reinforce the findings of some other investigations. For example, the challenges associated with an inadequate understanding and valuing of their role have previously been noted [21]. In a related vein, perspectives regarding these professionals’ scope of practice have previously been identified, whether related to the interpretation of this scope by physicians [17, 27] or registered nurses [17, 19] or to PHCNPs’ beliefs that their scope of practice is unduly limited [14, 16, 45]. The tension between the biomedical and nursing paradigms has been noted in some other investigations [13, 14, 19, 46]. Some other studies also have identified a lack of congruence between the reality of PHCNPs’ roles and the structures in which their practice is situated. Some examples, include the level of remuneration for these professionals’ services [14, 47] and the nature of their work schedule [18].

However, beyond this reinforcement or convergence with some previous studies, our study has highlighted some other elements that have emerged less clearly to date. A more precise image has emerged of the sense of fragility experienced by PHCNPs regarding their role. Among other elements, this fragility appears to be associated with the milieu- (in which clinic/s they work) and individual-dependent (with whom they specifically work) nature of the implementation of their role. This fragility also seems to be related to health system-level changes as various reforms have continued to be enacted (e.g., legislation requiring greater physician productivity).

As noted, the various specific elements that characterise the PHCNPs’ subjective experience of work appear to be grouped into two overriding themes: an inadequate understanding and valuing of their role, and concerns about their engagement in the work. However, beyond the clarity provided by these themes, our analysis suggests an overarching link between the meaning attributed by these professionals to their work and their sense of engagement in this work. The work context in which PHCNPs are required to practice can undermine this meaning of work and, in turn, can lead them to question their engagement in the work, or even within their profession.

By work meaning, we are referring to the sense of congruence experienced by individuals with the various elements upon which their work is constituted. Work is considered meaningful by individuals when the tasks and the context in which they are carried out are consistent with their identity [48]. The way in which PHCNPs describe their subjective work experience appears to reveal a significant gap between their expectations and their work reality, which in turn affects the meaning that they attribute to their work. This gap appears related to these professionals’ perception that their role is not recognised nor used to its’ full potential in the work teams. On the one hand, the dynamics of collaboration with the partner physicians and other professionals do not always permit the PHCNPs to fully utilise their skills. On the other hand, some of the structures in place appear to be poorly adapted to these professionals’ level of responsibility for which they could legitimately be held accountable. In addition, these professionals’ subjective work experiences appear to be influenced by their vulnerability in the face of changes that occur that are beyond their control both at the clinic (organisational) level or at the health care system level.

With respect to work engagement, we draw upon the concept of agency [40, 49] to conceptualise this notion as a practice situated in organisational and health system contexts with regards to the interrelationship between individuals (their expectations and aspirations for work; their life course), their profession (role, values, evolution of professional work), organisational characteristics (health organisations, professional and complex) and broader structural dimensions (evolution of the health system, gender inequality, professional hierarchies). In this conceptualisation, PHCNPs’ work engagement is closely tied to their hopes regarding their work and their profession, but equally to the practice context and to the broader evolution of the health care system. The frustrations and uncertainty being experienced by many PHCNPs seem to have led them to various forms of disengagement at work including a questioning of their future within their profession.

Consistent with this analysis, it seems clear that the optimisation of PHCNPs’ role at the patient care level is influenced by elements at the organisational and health system context levels. For example, these professionals’ frustrations regarding their remuneration and schedule are related to structures that have been put in place at other levels (e.g., collective agreement). Similarly, although the obstacles experienced in their collaboration with some physicians may be linked to some of these latter professionals’ reluctance to recognise the PHCNPs’ distinct role, they also seem to be at least partly explained by new productivity pressures that were imposed on physicians within the context of a specific health care reform.

The theoretical approach that we have used has helped to highlight that the PHCNPs have to deal individually with these structural constraints in their working environment. The context, that is, the *structural*, plays a role in constraining action, limiting the optimal use of the PHCNP role. These professionals feel isolated and sometimes poorly supported both by their professional licensing body and their union, which do not always appear to have taken into account their specific requirements. This situation may be exacerbated for those PHCNPs who are the sole member of this profession in their clinic. Depending on the specific milieu within which they work or the physicians with whom they work, the PHCNPs, whether alone or with colleagues, have few levers to increase their capacity for action individually or collectively in order that the work more closely resembles their representation of their profession and the nature of their contribution in the health system. These professionals must fall back on the relationship with their partner physician(s) as the key lever to optimise their capacity for action.

To achieve an optimal use of the PHCNP role, it appears necessary to work at various levels to enable these professionals to increase their agency. Focusing solely on individual competencies and personal attributes, which tends to be the emphasis, appears insufficient. A more profitable approach is likely to also include the establishment of appropriate work environments and congruent structures across organisational and health system context levels that favour the implementation and deployment of new professional roles and new modes of collaboration in interprofessional teams.

Several specific avenues have emerged from our investigation. Our findings have reinforced the importance, also noted by some other authors, of ensuring a uniform understanding of the PHCNP scope of practice [16, 50] and the accompanying role description [20, 21, 23]. It also seems clear that endeavours to optimise PHCNPs’ role must include better articulation between the individual, organisational and health system context levels. Our results also reveal the importance of fostering work environments that limit the individual- and milieu-dependent nature of PHCNPs’ role so that they can increase their agency in the workplace, positively influencing their sense of engagement. Thus, optimizing the role of PHCNPs refers to the possibility for these professionals to take on leadership roles within multidisciplinary teams, to be involved in executive team meetings within clinics and to potentially participate in regional and provincial forums. Finally, since we completed our data collection, new guidelines for physicians have been published in Quebec with respect to the PHCNP role [51]. It is clear that further research will be needed to evaluate the impact of this type of initiative in addressing the challenges that we have identified.

Regarding the limits of our investigation, the generalisability of the conclusions may be limited by our focus upon PHCNPs’ experiences in one jurisdiction (Quebec). Nevertheless, the broad trends to which these experiences appear to be linked (e.g., increased productivity pressures) suggest that our findings may have broader applicability [52]. The comprehensive nature of the individual and focus group interviews that were conducted as well as the ongoing validation of the findings carried out with the two committees over the course of the research lead us to feel confident that we have accurately understood some important trends despite the limited number of interviews in a single jurisdiction.

## Conclusions

The findings of this investigation have revealed the sense of fragility experienced by PHCNPs regarding their role. There appears to be an overarching link between meaning attributed by these professionals to their sense of engagement in this work. The optimisation of PHCNPs’ role requires coherent efforts across individual, organisational and health system context levels.

## Data Availability

The individual and focus group data that support the findings of this study are unavailable due to being potentially identifiable.

## Abbreviations

APN: Advanced Practice Nursing
CIUSSS-CN: Centre intégré universitaire de santé et de services sociaux de la Capitale-Nationale
DNS: Director of Nursing Services
NP: Nurse practitioner
PHCNP: Primary Health Care Nurse Practitioner
